# Identification of a homogenous structural basis for oligomerization by retroviral Rev-like proteins

**DOI:** 10.1186/s12977-017-0366-1

**Published:** 2017-08-22

**Authors:** Chijioke N. Umunnakwe, Karin S. Dorman, Drena Dobbs, Susan Carpenter

**Affiliations:** 1 Department of Animal Science, Iowa State University, Ames, IA 50011 USA; 2 Program in Bioinformatics and Computational Biology, Iowa State University, Ames, IA USA; 3 Department of Genetics, Developmental and Cell Biology, Iowa State University, Ames, IA USA; 4 Department of Statistics, Iowa State University, Ames, IA USA; 5 HIV Dynamics and Replication Program, Present Address: National Cancer Institute, 1050 Boyles St, Frederick, MD 21702 USA

**Keywords:** Retroviruses, Rev, Rev-like proteins, Coiled-coil, Ancestral inference, Helical wheel

## Abstract

**Background:**

Rev-like proteins are post-transcriptional regulatory proteins found in several retrovirus genera, including lentiviruses, betaretroviruses, and deltaretroviruses. These essential proteins mediate the nuclear export of incompletely spliced viral RNA, and act by tethering viral pre-mRNA to the host CRM1 nuclear export machinery. Although all Rev-like proteins are functionally homologous, they share less than 30% sequence identity. In the present study, we computationally assessed the extent of structural homology among retroviral Rev-like proteins within a phylogenetic framework.

**Results:**

We undertook a comprehensive analysis of overall protein domain architecture and predicted secondary structural features for representative members of the Rev-like family of proteins. Similar patterns of α-helical domains were identified for Rev-like proteins within each genus, with the exception of deltaretroviruses, which were devoid of α-helices. Coiled-coil oligomerization motifs were also identified for most Rev-like proteins, with the notable exceptions of HIV-1, the deltaretroviruses, and some small ruminant lentiviruses. In Rev proteins of primate lentiviruses, the presence of predicted coiled-coil motifs segregated within specific primate lineages: HIV-1 descended from SIVs that lacked predicted coiled-coils in Rev whereas HIV-2 descended from SIVs that contained predicted coiled-coils in Rev. Phylogenetic ancestral reconstruction of coiled-coils for all Rev-like proteins predicted a single origin for the coiled-coil motif, followed by three losses of the predicted signal. The absence of a coiled-coil signal in HIV-1 was associated with replacement of canonical polar residues with non-canonical hydrophobic residues. However, hydrophobic residues were retained in the key ‘a’ and ‘d’ positions, and the α-helical region of HIV-1 Rev oligomerization domain could be modeled as a helical wheel with two predicted interaction interfaces. Moreover, the predicted interfaces mapped to the dimerization and oligomerization interfaces in HIV-1 Rev crystal structures. Helical wheel projections of other retroviral Rev-like proteins, including endogenous sequences, revealed similar interaction interfaces that could mediate oligomerization.

**Conclusions:**

Sequence-based computational analyses of Rev-like proteins, together with helical wheel projections of oligomerization domains, reveal a conserved homogeneous structural basis for oligomerization by retroviral Rev-like proteins.

**Electronic supplementary material:**

The online version of this article (doi:10.1186/s12977-017-0366-1) contains supplementary material, which is available to authorized users.

## Background

Retroviruses such as HIV-1 require nuclear export of incompletely spliced and unspliced viral mRNA to generate viral proteins and genomic RNA for replication. Lentiviruses, deltaretroviruses and a few betaretroviruses encode a regulatory protein that mediates this essential step. Lentiviruses encode the regulatory protein Rev [1–9]; deltaretroviruses encode a Rev analog, Rex [10, 11]; and three betaretroviruses, mouse mammary tumor virus (MMTV), Jaagsitke sheep retrovirus (JSRV), and the human endogenous retrovirus type K (HERV-K HML2), encode the regulatory proteins Rem [12, 13], Rej [14], and Rec, respectively [15]. This family of proteins, collectively termed Rev-like proteins, is believed to share a similar mechanism of function despite being highly divergent at the sequence level.

HIV-1 Rev is the best characterized of the retroviral Rev-like proteins. Following translation, HIV-1 Rev traffics to the nucleus, binds a ~350 nucleotide target in the viral pre-mRNA, termed the Rev-responsive element (RRE), oligomerizes on the RRE, and associates with host export factors to form an export-competent complex that exits the nucleus [16, 17]. Specific motifs or domains in HIV-1 Rev and other Rev-like proteins mediate distinct steps of the Rev pathway, including nuclear import, RNA binding, oligomerization and nuclear export [18–21].

Early studies established that an arginine-rich motif (ARM) within the α-helical region of HIV-1 Rev recognizes a high affinity binding site within the HIV-1 RRE [22–26]. This initial binding of HIV-1 Rev to viral RNA is followed by assembly of additional Rev molecules along the RRE and recruitment of the host cellular factor CRM1 for nucleocytoplasmic export of the ribonucleoprotein complex [27–29]. More recent structural studies revealed a re-ordering of HIV-1 Rev dimers upon RRE binding, the result of plasticity in Rev oligomerization domains [30]. This plasticity could allow Rev to adopt a wide array of dimeric and multimeric conformations that facilitate interactions with a genetically variable and conformationally pliable RRE [31, 32].

Equine infectious anemia virus (EIAV) Rev is functionally homologous to primate lentivirus Revs, but is also unusual in several aspects. EIAV Rev is genetically variable, differs in the organization of functional domains [33], and contains a novel bipartite RNA binding domain (RBD) comprising two ARMs (ARM-1 and ARM-2) separated by 79 amino acids in primary sequence [34]. More recent findings also suggest that RNA binding requires coiled-coil interactions that mediate dimerization of EIAV Rev monomers, thereby bringing ARM-1 and ARM-2 into close proximity for RNA binding [35]. This is reminiscent of structural studies of HIV-1 Rev/RRE interactions, which suggest that dimerization of HIV-1 Rev juxtaposes the ARMs of each monomer at high affinity binding sites on the RRE [29, 36, 37]. Thus, while the bipartite RBD of EIAV Rev is novel among lentivirus Revs, it appears that dimerization and juxtaposition of Rev ARMs on the RRE could be a shared feature of lentiviral Rev-RRE interactions.

The possible parallels in the manner by which EIAV and HIV Rev associate with their cognate RNA elements raises the possibility that a coiled-coil motif might also exist within the oligomerization domain of HIV-1 Rev, and perhaps other retroviral Rev-like proteins. In the present study, we investigated the extent of structural homology among retroviral Rev-like proteins by undertaking a comprehensive computational analysis of overall protein domain architecture and specific secondary structural elements for representative members of the Rev-like family of proteins. Results demonstrated that a coiled-coil motif is likely an ancestral trait in retroviral Rev-like proteins and is still functional in HIV-1 Rev, where it is involved in dimerization and oligomerization. Coiled-coil or coiled-coil derived motifs were found associated with oligomerization domains of other retroviral Rev-like proteins, indicating that a common structural motif likely underlies essential protein–protein interactions in the Rev-like family of proteins.

## Results

### Rev-like proteins share a similar functional organization

**Fig. 1:**
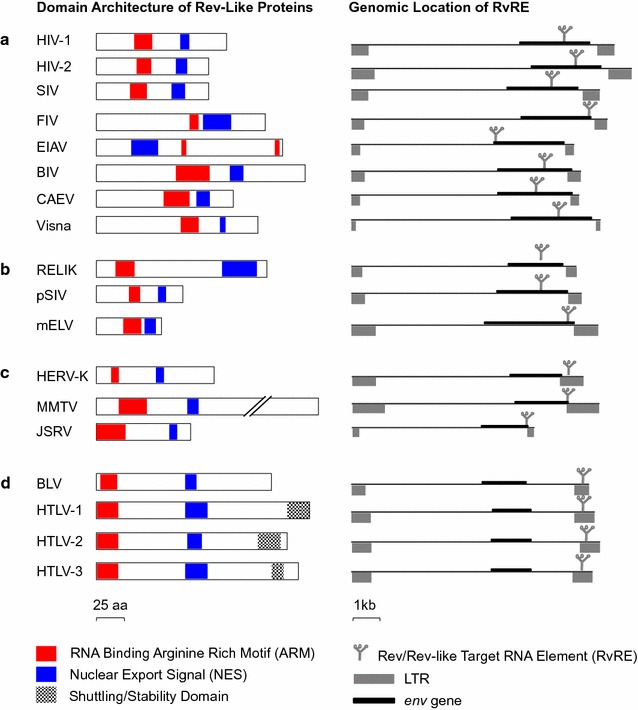
Domain organization of Rev-like proteins (*left*) and location of cognate RNA target elements (RvRE) in the proviral genome (*right*) in: **a** Exogenous lentiviruses; **b** endogenous lentiviruses; **c** betaretroviruses; **d** deltaretroviruses. *Red colored boxes* in protein schematics represent RNA binding arginine rich motifs (ARMs), *blue boxes* represent nuclear export signals (NES), and *checkered boxes* represent shuttling/stability domains unique to HTLV members. Stem loop structures in viral genomes (*right*) represent location of the RvRE target. LTRs are shown as *grey terminal boxes* and the *env* gene is shown as a *thick black rectangle* within the genome

To determine whether the overall architecture of Rev-like proteins follows a conserved structural pattern, we compared the organization of protein domains of representative Rev-like members of lentiviruses, deltaretroviruses and betaretroviruses. Although Rev-like proteins vary in length, a similar organization of protein domains in which the NES is located C-terminal to the ARM was observed in all members except EIAV Rev (Fig. 1, left). In addition, only the Rev-like proteins of human deltaretroviruses contained a stability/shuttling domain, proximal to the C-terminus (Fig. 1d) [38]. The RNA target elements of all Rev-like proteins, hereafter termed RvRE, are always found in the *env*/LTR region of the genome (Fig. 1, right). Lentiviral RvRE are typically located in the central region of *env*; however, the RvRE of EIAV is located at the 5′ end of *env*, while the RvRE of FIV and the endogenous ferret lentivirus, mELV, are located at the 3′ end of *env* (Fig. 1a, b). In beta- and deltaretroviruses, the RvRE is located near the start of or within the U3 region of LTR (Fig. 1c, d). Despite some variations, the overall organization of protein domains in retroviral Rev-like proteins and the location of target RNA elements within their cognate viral genomes revealed a similar structural organization. Interestingly, EIAV Rev is an outlier among Rev-like proteins with respect to the overall organization of functional domains and location of the RvRE. It is not clear if/how this may relate to some of the unique aspects of EIAV pathogenesis [33].

### α-Helical secondary structures are a common feature of Rev-like proteins

In HIV-1 Rev, a helix-turn-helix structural motif mediates intra- and intermolecular protein–protein interactions required for high affinity RNA binding and oligomerization [29, 37]. To determine whether other Rev-like proteins contain similar local structural topologies, we analyzed specific predicted secondary structural elements in representative retroviral Rev-like proteins.

**Fig. 2:**
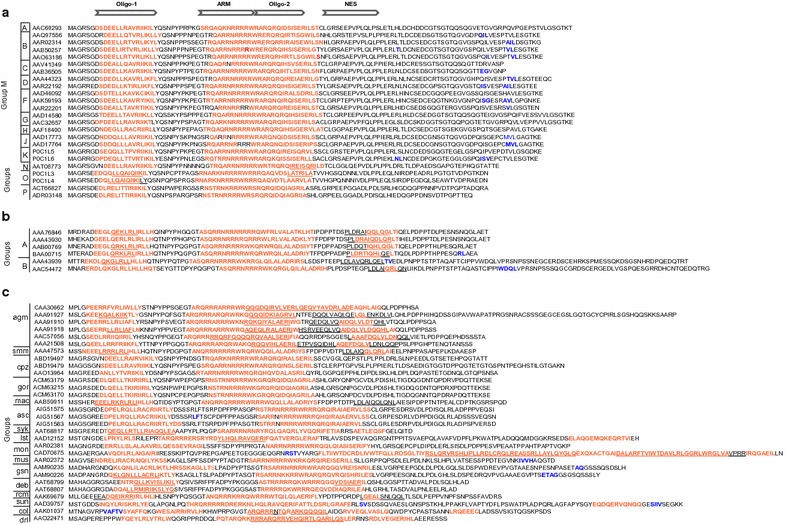
Distribution of α-helices and coiled-coil signals in representative Rev proteins of primate lentiviruses, **a** HIV-1, **b** HIV-2, and **c** SIV. Rev sequences, including accession numbers are ordered by groups, and HIV-1 group M is further ordered by clade, A–K. *White arrows* above all sequences indicate locations of the following Rev functional domains: N-terminal oligomerization domain (Oligo-1); C-terminal oligomerization domain (Oligo-2); RNA binding arginine rich motif (ARM); and nuclear export signal (NES). Residues in *red* indicate regions of predicted α-helices; residues in *blue* indicate predicted β-strand regions, and underlined residues represent regions of predicted coiled-coils. Sequences in the figure are a subset of the complete dataset in Additional file 2, which includes genome, Pol, and Env accession numbers for each sequence

Secondary structure predictions indicated that the two α-helices located in the N-terminal half of HIV-1 Rev (red residues, Fig. 2a) are conserved in Rev proteins of the other primate lentiviruses (Fig. 2b, c). The first helix encompasses the N-terminal oligomerization domain (Oligo-1) while the second helix overlaps the arginine-rich motif (ARM) and the downstream oligomerization domain (Oligo-2). Thus, the helix-turn-helix structure present in the N-terminal half of the HIV-1 Rev crystal structure [29] is a conserved structural feature of all primate lentivirus Rev proteins. Extended unstructured regions were present in the C-terminal half of HIV-1 and HIV-2 Revs and in most of the SIV Revs, although an additional short α-helical region, overlapping the NES, was predicted for some HIV-2 and SIV Revs (Fig. 2b, c).

At least two α-helical domains were also predicted for Rev-like proteins of non-primate lentiviruses and betaretroviruses (Additional file 1). Overall, however, these Rev-like proteins contained relatively more α-helical regions, and less unstructured regions, as compared to primate lentivirus Revs. Surprisingly, the deltaretrovirus Rev-like proteins contained little or no detectable ordered secondary structure (Additional file 1). The presence of intrinsically disordered regions in some retroviral Rev-like proteins may contribute to the structural flexibly recently described for HIV-1 Rev [17].

### Predicted coiled-coil motifs are widely distributed among Rev-like proteins

Coiled-coils are a subset of α-helices that mediate protein–protein interactions. In EIAV Rev, computational and biochemical studies indicated that a predicted coiled-coil motif in the central region of the protein is required for dimerization and RNA binding [35]. The CCHMM_PROF coiled-coil server [39], was used to assess whether coiled-coils were predicted for other retroviral Rev-like proteins. Predicted coiled-coil motifs were identified in the Rev proteins of all primate lentivirus species analyzed; however, their distribution varied within groups (Fig. 2, Additional file 1). Coiled-coils were predicted in all Rev sequences analyzed from HIV-2 Groups A and B, and from SIV African green monkey (agm), sooty mangabey (smm), and macaque (mac). In addition, some Rev sequences from SIV mona (mon), greater spot nosed monkey (gsn), as well as HIV-1 groups N and O were predicted to contain coiled-coils. In contrast, coiled-coils were not identified in any HIV-1 group M Rev sequences, in SIV chimpanzee (cpz), or red tailed monkey (asc). When present in the primate lentivirus Revs, the coiled-coils were usually localized within or overlapping oligomerization domains (Fig. 2), suggesting they may play a role in protein–protein interactions.

Coiled-coil motifs are also widely distributed in the Rev sequences of non-primate lentiviruses, with the exception of the small ruminant lentivirus (SRLV) group (Additional file 1). Two out of three betaretroviruses Rev-like proteins, MMTV Rem and HERV-K Rec, were predicted to contain coiled-coils (Additional file 1). As expected, predicted coiled-coils were absent in the deltaretrovirus Rex proteins, which were predicted to lack α-helixes (Additional file 1). Taken together, these results indicate that the coiled-coil motif is a common structural feature within specific α-helical regions of Rev-like proteins.

### Predicted coiled-coils segregate along phylogenetic lineages in primate lentiviruses

**Fig. 3:**
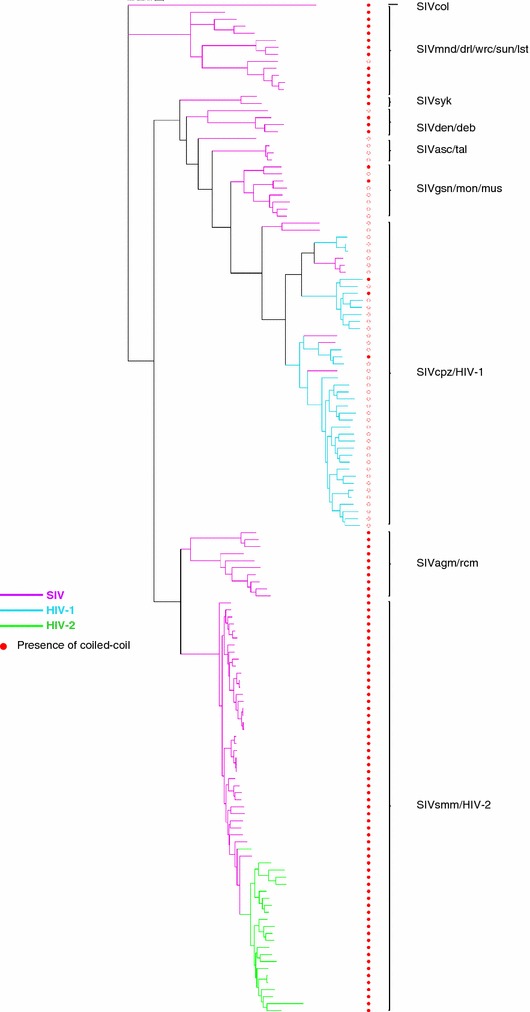
Presence of predicted Rev coiled-coils (*filled red circles*) in members of primate lentiviruses, SIV (*pink*), HIV-1 (*cyan*), and HIV-2 (*green*), in the context of an Env phylogenetic tree rooted with SIV colobus (SIVcol)

The differential distribution of coiled-coils in the Rev proteins of primate lentiviruses may reflect the phylogenetic lineages of HIV-1 and HIV-2. HIV-1 is derived from SIVcpz, which arose from recombination events resulting in a mosaic genome wherein *pol* is derived from red-capped mangabey (SIVrcm) and *env*/*rev* is derived from SIVgsn/mon/mus [40]. In contrast, HIV-2 is derived from SIVsmm. This evolutionary history suggested that the absence of coiled-coils in HIV-1 Revs reflects the absence of coiled-coils in their SIV ancestors. To test this hypothesis, the presence or absence of coiled-coils was mapped onto an Env-based phylogeny of primate lentiviruses. As shown in Fig. 3, HIV-1 and SIVcpz formed a monophyletic clade with the SIVgsn/mon/mus lineage that was largely bereft of coiled-coils (indicated by red circles). In contrast, HIV-2 and SIVsmm formed a monophyletic clade with the SIVagm/rcm lineage in which the coiled-coil signal was present in every member. From these results, we conclude that the presence/absence of coiled-coils in the Rev proteins of primate lentiviruses is a lineage-specific trait.

### Coiled-coil motifs are an ancestral feature of retrovirus Rev-like proteins

**Fig. 4:**
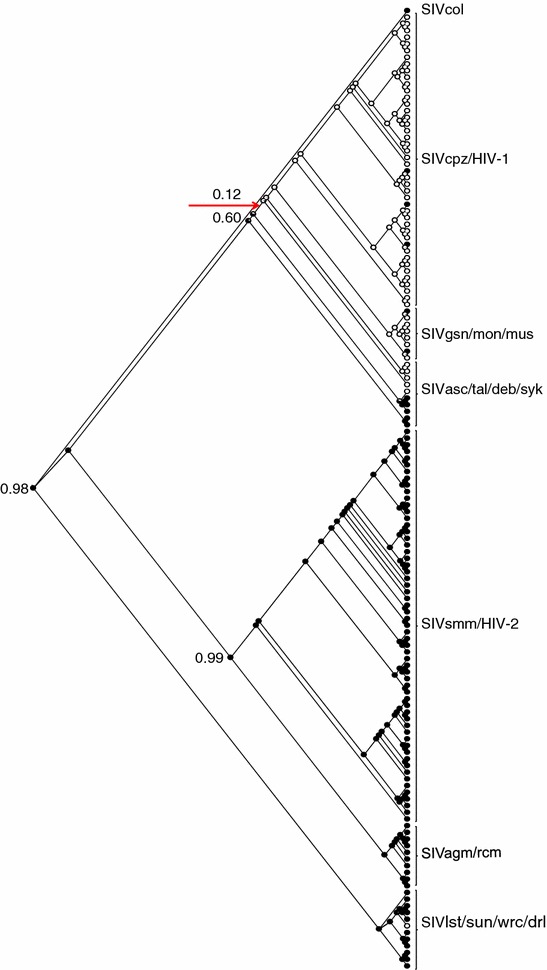
Ancestral inference of coiled-coils in Rev proteins of primate lentiviruses. Analysis was performed using the Env-based tree shown in Fig. 3. *Black area of filled circles* indicates the probability of Rev coiled-coils being present at that node; *red arrow points* to a single major transition from higher to lower probability of the coiled-coil signal in the phylogeny (0.60–0.12 probability). The probability of a Rev coiled-coil in the ancestral node of SIVsmm/HIV-2 and SIVagm/rcm (0.99), and the overall probability of an ancestral Rev coiled-coil for all lentiviruses (0.98) are indicated

The identification of coiled-coils in Rev-like proteins of divergent retroviruses combined with their phylogenetic distribution in primate lentiviruses, indicated that coiled-coils may be an ancestral feature of retroviral Rev-like proteins. To explore this idea, the ancestral state of the coiled-coils was modeled on the Env-based phylogeny of primate lentiviruses depicted in Fig. 3. Ancestral inference by maximum likelihood yielded a 0.98 probability that the ancestral Revs of all primate lentiviruses contained a coiled-coil motif (Fig. 4). The analysis also indicated one major loss of the coiled-coil signal in the primate lentiviruses (Fig. 4, red arrow), resulting in the absence of coiled-coils in SIVcpz/HIV-1 group.

**Fig. 5:**
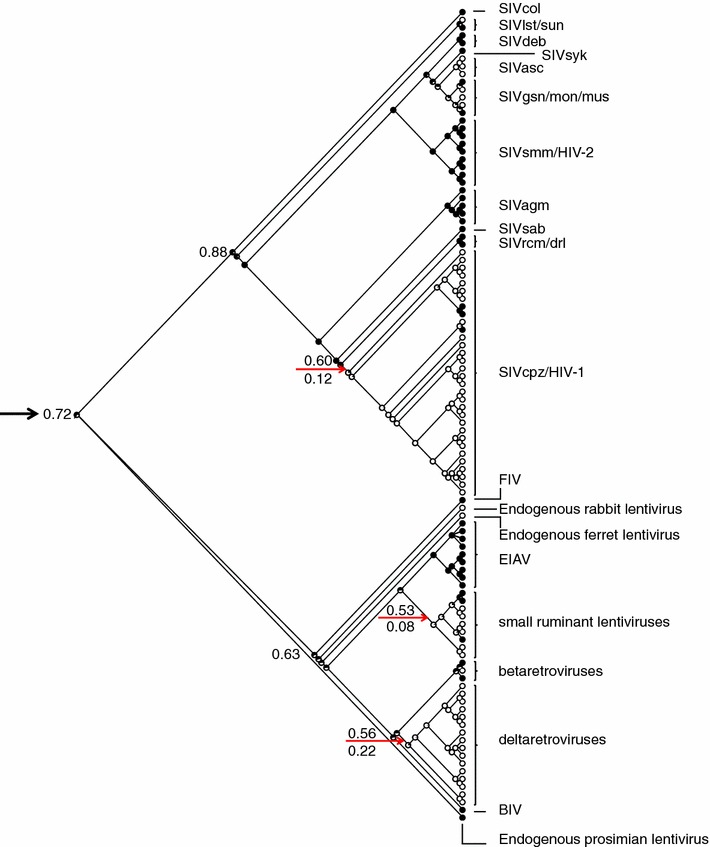
Evolutionary history of coiled-coil structure in retroviruses encoding Rev-like proteins using a Pol-based tree. *Area of filled circles* indicates probability of coiled-coils being present at that node; *red arrows* correspond to major transitions from high to low probability of coiled-coils signals in the phylogeny. *Black arrow* indicates the overall probability of an ancestral Rev coiled-coil

Having obtained strong evidence that the coiled-coil phenotype is an ancestral feature in primate lentivirus Revs, we expanded our analyses to include all retroviral members that contain Rev-like proteins. The ancestral state of coiled-coils was modeled on a Pol-based phylogeny of all retroviruses encoding Rev-like proteins (Fig. 5). Ancestral inference by maximum likelihood yielded a 0.72 probability that the coiled-coil signal was present in the ancestral sequence to all retroviral members that encode Rev-like proteins (Fig. 5, black arrow). The Pol-based tree depicts two groups, one comprising all primate lentiviruses and the other comprising all other Rev-like encoding retroviruses. In addition to the loss of the coiled-coil signal in the SIVcpz/HIV-1 lineage of the primate lentivirus group (Fig. 5, top red arrow), there were two additional losses in the group comprising all other Rev-like encoding retroviruses: one occurring on the branch leading to the small ruminant lentiviruses, and the other occurring on the branch leading to the deltaretroviruses (Fig. 5, bottom two red arrows). These losses are reflected in the absence of coiled-coil signals in the small ruminant lentivirus and deltaretrovirus groups (see Additional files 1, 2).

To ensure that the absence of coiled-coils in the SIVcpz/HIV-1, SRLVs, and deltaretrovirus groups was not due to a bias in the prediction algorithm used, we reanalyzed all sequences for coiled-coil motifs using another coiled-coil server, COILS, which utilizes a different prediction method [41]. The overall correlation between predictions from the two different servers was 0.68, and the two servers agreed on the absence of coiled-coils in the SIVcpz/HIV-1, SRLVs, and deltaretrovirus groups (Additional file 2). Thus, these analyses provided additional support that coiled-coils are an ancestral structural motif of retroviral Rev-like proteins.

### The loss of a coiled-coil signal in HIV-1 Rev is associated with genetic variation in the canonical coiled-coil sequence motif

**Fig. 6:**
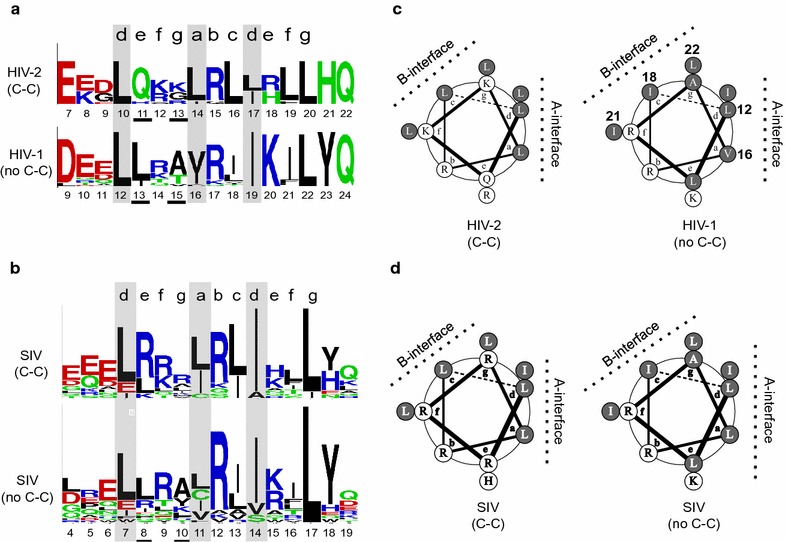
Comparison of N-terminal oligomerization domains of primate lentivirus Revs. **a** Sequence logo of HIV-1 and HIV-2 Rev, depicting coiled-coil (C-C) registers predicted for HIV-2 and the corresponding region in HIV-1 Rev sequences predicted not to contain coiled-coils (no C-C). *Shaded outlines* indicate ‘*a*’ and ‘*d*’ core registers, *underlined residues* indicate major genetic differences between HIV-1 and HIV-2. *Colored residues* indicate polar residues and *black residues* indicate hydrophobic residues; *red* and *blue colored residues* represent polar charged residues, and *green colored* residues represent polar non-charged residues. **b** Sequence logo depicting coiled-coil registers predicted for a subset of SIV Rev sequences predicted to contain coiled-coils and the corresponding region in SIV Rev sequences predicted not to contain coiled-coils. *Shaded outlines* indicate ‘*a*’ and ‘*d*’ core registers and *underlined residues* indicate sites of genetic differences. *Colored residues* indicate polar residues and *black residues* indicate hydrophobic residues; *red* and *blue colored* residues represent polar charged residues, and *green colored* residues represent polar non-charged residues. **c** Helical wheel diagrams of the coiled-coil region of HIV-2 Rev and the corresponding region in HIV-1 Rev. *Filled circles* indicate hydrophobic residues and *dashed lines* indicate predicted interaction interfaces, designated A-interface or B-interface. *Numbered residues* on the helical wheel of HIV-1 indicate residues known to mediate dimerization and oligomerization in published HIV-1 crystal structures [29, 37]. **d** Helical wheel diagrams of the coiled-coil region for the subset of SIV Revs predicted to contain coiled-coils, and the corresponding region in SIV Revs predicted not to contain coiled-coils. *Filled circles* indicate hydrophobic residues and *dashed lines* indicate two predicted interaction interfaces, designated A-interface or B-interface

Prediction of coiled-coil motifs in a given protein is based on sequence features present in well characterized canonical coiled-coils [42]. Retroviral sequences are highly variable, and the absence of predicted coiled-coil signals in certain lineages such as SIVcpz/HIV-1 could be due to genetic variation that weakens the designation criteria. To explore this possibility, we analyzed genetic variation within the N-terminal oligomerization domains overlapping the predicted coiled-coils of primate lentiviruses and compared the sequence logos of groups that have a strong coiled-coil signature with groups that do not (Fig. 6). The HIV-2 Rev oligomerization domain displays hydrophobic residues in core ‘*a*’ and ‘*d*’ registers of an ‘*abcdefg*’ heptad motif, which is characteristic of canonical coiled-coils (Fig. 6a). Charged and polar residues predominate at the ‘*e*’ and ‘*g*’ positions, which again is consistent with the canonical coiled-coil formulation [43]. Interestingly, HIV-1 Rev also contained hydrophobic residues in the core ‘*a*’ and ‘*d*’ registers, conforming to the basic signature of a coiled-coil (Fig. 6a, shaded). The major sequence differences between HIV-1 and HIV-2 Rev are in the first ‘*e*’ and ‘*g*’ positions of the ‘*abcdefg*’ heptad motif (Fig. 6a, underlined), where HIV-1 Rev contains hydrophobic residues rather than the polar/charged residues found in HIV-2 and other canonical coiled-coils. The presence of hydrophobic residues at these sites may explain why coiled-coils are not predicted for most HIV-1 Rev sequences, as this runs counter to the canonical coiled-coil formulation.

Comparison of SIV Rev sequences that differ with respect to presence/absence of coiled coils showed a mixed population of hydrophobic and charged residues at the first ‘*e*’ and ‘*g*’ positions (Fig. 6b, underlined). Charged residues predominate in SIV Revs predicted to contain a coiled-coil, whereas hydrophobic residues predominate in SIV Revs predicted to lack a coiled-coil. It is likely, therefore, that genetic variation and selection for additional hydrophobic residues in the N-terminal oligomerization domain weakened the coiled-coil signal in Rev proteins of some primate lentiviruses, including HIV-1 Rev.

### Coiled-coil configurations are located at dimerization and oligomerization interfaces of HIV-1 Rev

The presence of hydrophobic residues in the ‘*a*’ and ‘*d*’ registers of both HIV-1 and HIV-2 Rev suggested that the N-terminal oligomerization domains of HIV-1 could adopt a coiled-coil structure similar to that of HIV-2 Rev. Indeed, helical wheels generated from HIV-1 and HIV-2 Rev show a high degree of similarity in this region, with each having two interaction interfaces, designated A-interface and B-interface (Fig. 6c, dotted lines). However, the interaction interfaces of HIV-1 Rev are more hydrophobic due to the additional hydrophobic residues in the first ‘*e*’ and ‘*g*’ registers. Helical wheels generated from SIVs Revs with and without predicted coiled-coil motifs also revealed two interacting interfaces, and the interaction interfaces of SIVs not predicted to contain coiled-coils were more hydrophobic than SIVs predicted to contain coiled-coils (Fig. 6d).

To infer the functional significance of these interfaces, we compared residues at the predicted interfaces with residues found to mediate dimerization and/or oligomerization in two available HIV-1 Rev crystal structures [29, 37]. In the Daugherty structure, L12 and V16 mediate dimerization, while residues I18, I21, and L22 mediate oligomerization. In our helical wheels, L12 and V16 are located at the A-interface, while I18, I21 and L22 are located at the B-interface (Fig. 6c). These two predicted helical interfaces also mediate dimerization and oligomerization in the DiMattia et al. crystal structure of HIV-1 Rev (not shown). These analyses indicate that the N-terminal oligomerization domain of HIV-1 Rev can be configured as a coiled-coil like interface that mediates dimerization and oligomerization.

### Signatures of coiled-coil interfaces are found in most retrovirus Rev-like proteins

**Fig. 7:**
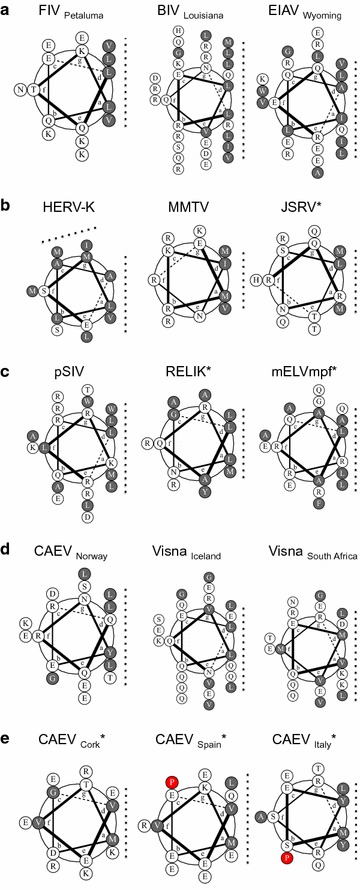
Helical wheel diagrams of predicted coiled-coil regions and analogous regions not predicted to contain coiled-coils for Rev-like proteins of: **a** feline, bovine, and equine lentiviruses; **b** betaretroviruses; **c** endogenous prosimian, rabbit, and ferret lentiviruses; **d**, **e** small ruminant lentiviruses. *Asterisks* indicate Rev-like proteins predicted not to contain a coiled-coil and the region analogous to their coiled-coil predicted counterparts. *Filled circles* indicate hydrophobic residues and *dashed lines* indicate potential interaction interfaces

Our analyses indicated that oligomerization of HIV-1 Rev is likely mediated by a coiled-coil motif and it was of interest to determine if coiled-coils could play similar roles in other Rev-like proteins. To investigate this, we extended our analysis of helical wheel interfaces to include predicted and potential coiled-coils of representative Rev-like proteins from each group of Rev-like encoding retroviruses (Fig. 7). The predicted coiled-coil regions in the lentiviruses BIV, FIV and EIAV each form a single hydrophobic interface comprising five or six hydrophobic residues (Fig. 7a). The predicted coiled-coil regions in betaretrovirus Rev-like proteins formed coiled-coil like helical wheels with at least one hydrophobic interaction interface (Fig. 7b). Of note, the analogous region of JSRV Rev, which was not predicted to contain a coiled-coil could also be configured into a helical wheel with an interaction interface similar to the betaretrovirus members predicted to contain coiled-coils. The Rev proteins of endogenous lentiviruses (Fig. 7c) also contained interaction interfaces that follow a pattern similar to that seen in FIV, BIV, EIAV (Fig. 7a) and the betaretroviruses (Fig. 7b). Each of the SRLV Revs with a predicted coiled-coil motif had an interaction interface containing a mix of hydrophobic and polar residues (Fig. 7d). In contrast, most SRLV Revs predicted to lack a coiled-coil have proline residues, which are helix breakers, in the analogous region (Fig. 7e). Similar to what we observed for Revs of primate lentivirus lineages, weakened or eliminated coiled-coil signals as a result of genetic variation correlated with loss of the coiled-coil signal in some Revs of the SRLV lineage.

Apart from HIV-1 Rev, few structural studies have been performed on Rev-like proteins. Therefore, there is little biophysical data documenting intermolecular interactions mediated by the interfaces predicted for most of the Rev-like proteins shown in Fig. 7. Molecular genetic studies, however, have provided some evidence that the predicted interfaces play a role in oligomerization of the respective Rev-like proteins. We previously reported that residues in the predicted EIAV Rev interface are critical for dimerization and RNA binding [35]. Both HERV-K Rec and SRLV Rev are known to oligomerize, and deletion of residues overlapping the predicted interaction interface in CAEV was found to abrogate dimerization [44]. When considered with available experimental data, our findings indicate that, with the exception of deltaretroviruses, oligomerization in retroviral Rev-like proteins is likely mediated by coiled-coil or coiled-coil-like structural motifs.

## Discussion

Dimerization and oligomerization of HIV-1 Rev along the RRE is an essential prerequisite for RNA export and viral replication [45]. In HIV-1 Rev, distinct dimerization and oligomerization domains mediate Rev assembly on the RNA target [17, 27]. Our previous studies of EIAV Rev identified a coiled-coil motif in the predicted oligomerization domain, which was suggested to mediate dimerization [35]. Here, we identified and characterized coiled-coils in Rev-like proteins from diverse retroviral genera. Coiled-coils were found to be an ancestral trait that largely persists in extant retroviruses, but whose signal is attenuated along certain phylogenetic lineages. For example, within primate Rev proteins of the lentivirus genera, the coiled-coil signal was dependent on HIV/SIV ancestry: Rev proteins descending from the HIV-2/SIVsmm lineage contain strong coiled-coil signals, whereas those from the HIV-1/SIVcpz lineage do not. Despite the lack of a predicted coiled-coil signal, the N-terminal oligomerization domain of HIV-1 Rev could be configured as a helical wheel with two hydrophobic interfaces, each containing residues shown in crystal structures to mediate dimerization or oligomerization of HIV-1 Rev. Similar helical wheels could be configured for most Rev-like proteins, with the exception of those in deltaretroviruses, which lack predicted secondary structure. The prevalence of coiled-coil signals across highly divergent Rev-like proteins, combined with the evolutionary patterns in their distribution and the observation that a coiled-coil-like motif mediates oligomerization in HIV-1 and EIAV Rev, indicate that the coiled-coil motif constitutes an ancestral and homogeneous mechanism of oligomerization in retroviral Rev-like proteins.

Sequence-based computational prediction of coiled-coil signals is generally reliable because coiled-coils have common and well-characterized features [46]. Sequences with residues that conform to the canonical coiled-coil motif are easily identifiable by coiled-coil prediction servers. However, coiled-coil like sequences that deviate from the canonical formulation by a few residues, especially in core registers of the coiled-coil sequence, can be predicted to have weak or no coiled-coil signals. A complementary, structure-based method to identify potential coiled-coil motifs is to represent sequences as helical wheels and compare their interfaces with those of known or predicted coiled-coils. When such analyses were applied to Rev sequences from the HIV-1/SIVcpz lineage, the structure-based signature of a coiled-coil was present, even though the sequences do not perfectly conform to the canonical coiled-coil signature. The protein–protein interfaces formed in the helical wheel configurations of HIV-1 Rev contain the specific residues shown to be involved in HIV-1 Rev dimerization and oligomerization in available crystal structures [29, 37]. Thus, Rev proteins in the HIV-1/SIVcpz lineage likely adopt a functional coiled-coil structure, even though a coiled-coil sequence signal was not readily identifiable by prediction servers.

Canonical coiled-coil motifs typically contain a combination of hydrophobic and hydrophilic residues that constrain oligomerization interactions in a characteristic manner [43, 46]. The coiled-coil-like motif identified here in HIV-1 Rev differs from canonical coiled-coils, such as those found in HIV-2 Rev or EIAV Rev, by the presence of hydrophobic residues at positions that typically contain polar residues. The additional hydrophobic residues in HIV-1 Rev not only mask the coiled-coil sequence signal, but also give rise to a more hydrophobic interface. The potential selective advantage of a more hydrophobic interface is not known; however, it could increase the variety of available hydrophobic interactions and consequent oligomeric conformations, thereby enhancing the plasticity of HIV-1 Rev. Interestingly, a major theme emerging from recent HIV-1 Rev-RRE-CRM1 structural studies is the plastic nature of the individual components of the ternary complex, especially Rev [17]. The plasticity of HIV-1 Rev oligomerization and RNA binding domains gives rise to a wide range of Rev binding conformations that can interact with different RRE structural conformations and influence functional activity [17, 31, 47]. Increased plasticity resulting from increased hydrophobicity may explain the loss of the canonical coiled-coil sequence signal, but not the structural motif, in the HIV-1/SIVcpz lineage of Rev proteins.

Presumably, the loss of the coiled-coil motif in deltaretroviruses and in some small ruminant lentivirus is also accompanied by a concomitant selective advantage. Modeling of deltaretrovirus Rex proteins (not shown) yields a globular protein with an extremely high degree of disorder and flexibility. Analogous to what we suggest for HIV-1 Rev, this increased flexibility could enable HTLV-1 Rex to bind diverse RRE structures. This hypothesis is supported by the observation that HTLV-1 Rex can functionally replace HIV-1 Rev, while the reverse is not true [48]. Within SRLV Rev proteins, the absence of the coiled-coil signal is often associated with the presence of a helix-breaking proline residue. This interruption in the helical structure most likely results in a more flexible region and could also contribute to plasticity of the protein. In support of this, the predicted structure of these SRLV Rev proteins yields a globular molecule with flexible loops, reminiscent of predicted protein structures of deltaretrovirus Rex proteins (not shown). Interestingly, there is regular cross-species transmission between goat and sheep lentiviruses [49], and the increased flexibility in some SRLV Rev might facilitate interaction with RRE targets from both ovine and caprine lineages. In most cases, therefore, the loss of coiled–coiled signals in retroviral Rev-like proteins is associated with predicted structural changes that would increase structural plasticity and expand the repertoire of potential Rev-RRE interactions.

## Conclusions

Analyses of sequence-based computational predictions of retroviral Rev-like proteins in a phylogenetic context identified an ancestral coiled-coil motif that is maintained across all retroviral genera, with the exception of deltaretroviruses. Helical wheel projections of HIV-1 Rev and other retroviral Rev-like proteins revealed interaction interfaces that could mediate oligomerization. When considered together, these complementary approaches suggest that the coiled-coil motif constitutes an ancestral and homogeneous mechanism of oligomerization in retroviral Rev-like proteins. Results from this study could inform strategies that seek to target Rev oligomerization and inhibit HIV RNA export and replication.

## Methods

### Domain architecture of Rev-like protein and genomic location of their RNA targets

Residue or nucleotide boundaries of arginine rich motifs, nuclear export signals, and Rev RNA targets of representative Rev/Rev-like proteins were mapped onto the corresponding protein/genomic schematic for each member. All schematics were represented according to scale. Accession codes used as references to construct schematics are as follows: HIV-1: K03455; HIV-2: U27200; SIV: X07805; FIV: M36968; EIAV: AF028232; BIV: M32690; CAEV: EU293537; Visna: L06906; HERV-K (HML-2): AAF88167; MMTV: M15122 and ABB02515; BLV: FJ914764; HTLV-1: U19949; HTLV-2: M10060; HTLV-3: DQ093792. The RELIK Rev sequence and motif boundaries used were described in [50], and sequences and motif boundaries for the endogenous lentiviruses mELV and pSIV were described in [51], and [52], respectively.

### Prediction of secondary structural elements

Secondary structure predictions for all Rev-like proteins were obtained using the JPred 3 server (http://www.compbio.dundee.ac.uk/jpred/index.html) [53]. JPred 3 provides a 3-state prediction (alpha-helix, beta-strand, other) with an accuracy of 81.5% [53]. Coiled-coil motif predictions were performed using both the CCHMM_PROF (http://gpcr.biocomp.unibo.it/cgi/predictors/cchmmprof/pred_cchmmprof.cgi) and COILS (http://www.ch.embnet.org/software/COILS_form.html) webservers [39, 41]. For CCHMM_PROF, default parameters were used; for COILS, the MTIDK matrix was used, with all window lengths, and with 2.5-fold weighting of positions ‘*a*’ and ‘*d*’ of coiled-coil repeats. All results of coiled-coil predictions for each of the Rev-like sequences used in this study are listed in Additional file 2.

### Phylogenetic reconstruction

Phylogenetic trees were constructed by Bayesian inference with MrBayes [54] v3.2 (http://mrbayes.sourceforge.net/download.php) using the rtRev amino acid substitution matrix, developed specifically for retroviral sequences [55]. The primate lentivirus Env tree (Fig. 3) was derived from an alignment of 144 Env sequences using the 2014 HIV Sequence Compendium [56] as a reference for collecting all HIV/SIV sequences. The Pol tree (Fig. 5) was derived from an alignment of 105 retroviral Pol amino acid sequences, and used to infer the overall ancestral state of coiled-coils in retroviral Rev-like proteins. Sequences used in the tree included sequences of publicly available retroviruses that encoded a Rev-like protein for which Pol and Env amino acid sequences were also available. Accession codes for all sequences included in all phylogenetic analyses are listed in Additional file 2. MrBayes analysis was run for 5,000,000 generations, sampling trees every 1000 generations and discarding the first 25% of samples as the burn-in fraction, as suggested by the authors [54]. Two Bayesian chains were run to ensure adequate mixing. Convergence was indicated by an average standard deviation of split frequencies (ASDSF) <0.05 between the two chains and a potential scale reduction factor (PSRF) value ~1 for all parameters. The 50% majority consensus tree was selected as the final tree. MrBayes analyses were run on the XSEDE cluster using the CIPRES Science Gateway for inference of large phylogenetic trees [57] (https://www.phylo.org/) with the BEAGLE library enabled. The Env tree in Fig. 3 was visualized using the iTOL webserver [58] (http://itol.embl.de/).

### Ancestral inference

Ancestral state reconstruction was performed with the MESQUITE software v3 [59] (https://mesquiteproject.wikispaces.com/) using both parsimony and maximum likelihood methods on each of the phylogenetic trees constructed. For all ancestral inferences performed, the predicted coiled-coil phenotype was modeled as a discrete trait. Maximum likelihood was performed using the “Markov k-state 1 parameter” model (MK1), a generalization of the Jukes–Cantor model in which there is a single parameter, the rate of change, and both forward (gain) or backward (loss) changes are equally likely.

### Sequence alignment, logos, and helical wheels

Sequence alignments were performed with the MAFFT webserver [60] (http://mafft.cbrc.jp/alignment/server/) using default parameters. Sequence logos were generated using the WebLogo server [61] (http://weblogo.berkeley.edu/logo.cgi), and helical wheels were obtained by using the DrawCoil 1.0 webserver (http://www.grigoryanlab.org/drawcoil/).

## Additional files

**Additional file 1.** Distribution of α-helices and coiled-coil structure in representative Rev-like proteins in (A) non-primate lentiviruses, (B) betaretroviruses, and (C) deltaretroviruses. Residues in red indicate regions of predicted α-helices; residues in blue represent predicted β-strand regions, and underlined residues represent regions of predicted coiled-coils.

**Additional file 2.** Complete dataset for sequences used in this study. For each SIV, HIV-1, HIV-2, EIAV, FIV, SRLV, BIV, BLV, PTLV, HERVK, MMTV, Jaagsiekte, and endogenous lentivirus Rev-like sequence, accession code information for the genome from which it was derived as well as Pol, Env, and Rev proteins are provided, where available. Results for the prediction of coiled-coils by CCHMM_PROF (http://gpcr.biocomp.unibo.it/cgi/predictors/cchmmprof/pred_cchmmprof.cgi) and COILS webservers (http://www.ch.embnet.org/software/COILS_form.html) [39, 41] are indicated for each sequence, and the overall correlation between the two servers for all sequences, as well as the correlation for a smaller subset comprising BLV, PTLV, SIV, and HIV-1 are also included.
