# Supplementary material for: Identification of a homogenous structural basis for oligomerization by retroviral Rev-like proteins
Source: Retrovirology. 2017 Aug 22;14:40. doi: 10.1186/s12977-017-0366-1 (PMC5568270; doi:10.1186/s12977-017-0366-1)
Supplement: Supplementary file 1 — Additional file 1. Distribution of α-helices and coiled-coil structure in representative Rev-like proteins in (A) non-primate lentiviruses, (B) betaretroviruses, and (C) deltaretroviruses. Residues in red indicate regions of predicted α-helices; residues in blue represent predicted β-strand regions, and underlined residues represent regions of predicted coiled-coils. [file 12977_2017_366_MOESM1_ESM.pdf]

A. Non-primate lentiviruses

FIV

AAB28971 MAEGGFTHNQQWIGPEEAEELLDFDIAVQMNEEGPLNPGVNPFRVPGITSQEKDDYCKILQTKLQELKNEVKEVKIEEGNAGKRKRQRRRRKKKAFKHMMANLENRFKMLFGTPSTTDETEEKTSAKEKRVDWEDYWDPEEIEKMLMD
AAB22932 MAEGFAANRQWIGPEEAEELLDFDKATQMNEEGPLNPGVNPFRVPAVTEADKQEYCKILQPRLQEIRNEIQEVKLEEGNAGKMKKRQRRRRKKKAFKKMMTDLEDRFRKLFGSPSKDEYTEIEIEEDPPKKEKRVDWDEYWDPEEIERMLMD
P20885 MAEGFAANRQWIGLEEAEELLDFDIATQMSEEGPLNPGVNPFRVPGITEKEKQNYCNILQPKLQDLRNEIQEVKLEEGNAGKRKRQRRRRKKKAFKRMMTELEDRFRKLFGTTSTTGDSTVDSEDEPPKKEKRVDWDEYWNPEEIERMLMD

EIAV

AAC24025 MAESKEARDQEMNLKEESKEEKRRNDWWKIGPQGPLGSDQWCRVLRQSLPEEKISSQTCIARRHLGPGPTQHTPSRRDRWIRGQILQAEVLQERLEWRIRGVQQVAKELGEVNRGIWRELHFREDQRGDFSAWGDYQQAQERRWGEQSSPRVLRPGDSKRRRKHL
AFW99174 MAEGRDSRYQEEMIPKEESKGKEEKGRNDWWKIAPQTPLDNDDWCWVLRQSLPEEKTPSQTCIARRALGPGPVQSTPSKRERWLRGKIQQAESLQEQLEWRIRGVQQSAEALREVNQGIWKELQWTRRLRGDYSSFYSSKREERRWGEESKPRILKPGDSKRRRKHL
AFW99186 MAEGRDSRYQEEMIPKEESKGKEEKGRKDWWKIAPQTPLDNDDWCWILRQSLPEEKTPSQTCIARRALGPGPVQSTPSKRERWLRGQIQQAESLQEQLEWRIRGVQQSAEALRKVNQGIWKELQWTRQLRGDYSSFYSSKREERRWGEESKPRILKPGDSKRRRKHL
AFW99180 MAEGRDSRYQEEMIPKEESKGKEEKGRNDWWKIAPQTPLDNDDWCWVLRQSLPEEKTPSQTCIARRELGPGPVQSTPSKRERWLRGQIQQAESLQEQLEWRIRGVQQSAEALRKVNQGIWKELQWTRQLRGDYSSFYSSKREERRWGEESKPRILKPGDSKRRRKHL
AFW99168 MAEGRDSRYQEEMIPKEESKGKEEKGRNDWWKIAPQTPLDNDDWCCILRQSLPEEKTPSQTCIARRELGPGPVQSTPSKRERWLRGQIQQAESLQEQLEWRIRGVQQSAEALREVNQGIWKELQWTRRLRGDYSSFYSSKREERRWGEESKPRILKPGDSKRRRKHL
ADU02711 MAEARDTRYQEEMNRKEEKEDNKRRNNWWKIDPQRPLDNDEWCRILRQSLPEEKIPSQTCIARRHLGPGPVSYVPGRRDSWLRGQVQHAEALQEQLEWRIRGVQQTAKELEKVNKEIWRELQYTRRQHGDYRSFGDYRRREEERWGESSPRVLKPGDSKRRRKHL
ADK35849 MAEARDTRYQEEMNRKEEKEDNKRRNNWWKIDPQRPLDNDEWCRILRQSLPEEKIPSQTYIARRHLGPGPVSCVPGRRDSWLRGQVQHAEALQEQLEWRIRGVQQTTKELEKVNKEIWRELQYTRRQHGDYGSFGDYRRREEERWGESSPRVLKPGDSKRRRKHL
ADK35808 MAEARDTRYQEEMNRKEDKEDKRRNNWWKIDPQRPLDNDEWCRILRQSLPEEKVPSQTCIARRHLGPGPVSCIPGRRDSWIRGQVQHAEVLQEQLKWRIRGVQQTAKELEKVNKEIWRELQYTRRQHGDYGSFCDYRRREEERWGESSPRVLKPGDSKRRRKHL
ADU02638 MAEARDTRYQEEMNRKEDKEDKRRNNWWKIDPQRPLDNDEWCRILRQSLPEEKVPSQTCIARRHLGPGPVSCVPGRRDSWLRGQVQHAEALQEQLEWRIRGVQQTAKELEKVNKGIWRELQYTRRQHGDYGSFDNYRRREEERWGESSPRILKPGDSKRRRKHL

BIV

AAA42772 MDQDLDRAERGERGGGSEELLQEEINEGRLTAREALQTWINNDSPRYVKKLRQGQPELPTSPGGGGGRGHRARKLPGERRPGFWKSLRELVEQNRRKQERRLSGLDRRIQQLEDLVRHMSLGSPDPSTPSASVLSVNPPAQTPLGHLPPRSYFKLKRVDCGAGWDLRTTAAPGLPICELDWIQGTK
AAA64393 MMEEGRKEEPEERGEKSTMRDLLQRAVDKGHLTAREALDRWTLEDHGPPVHHVKLGRSNTVSIAECARGYRPCRGRRPARRPPIRRHPSFWGTLRGLVSEAQRRQEDRMSDLENRMAELEERFEDLALVDSGGKNPAAPAQSVSPPSSNPFAYSLSHFSKSKRVDCGEKGNRWGRPGAFPGAGISELDWIESGDGGDERPKGGRYPRGGNTPS

SRLV

VISNA
AAA48357 MASKESKPSRTTRRGMEPPLRETWNQVLQELVKRQQQEEEEQQGLVSGLQASKADQIYTGNSGDRSTGGIGGKTKKKRGWYKWLRKLRAREKNIPSQFYPDMESNMVGMENLTLETQLEDNALYNPATHIGDMAMDGREWMEWRESAQKEKRKGGLSGQRTNAYPGK
AAA66816 MASSKNMPSRITQKSMEPPLRETWQQVVQEMVMRKQRDEEEKQNLVTGLQASSGDPIYTGNSSDRSTRGPGGKTKRRKGWFQWLRKLRAREKNIPSQFYPDMEGNCAGLENLTLGEGMEENPIYDTSAATNTANMDGRNWMEWR

CAEV
AEF12563 MDCGAREIHWTGREQWVEVNMEEEPLLGKQQRQGKYRKKGVQDIGYPHIPKGDHNNGSKTKRRKRNRGWWRRLRGVIRAGTTIQETNPDRSLEDCREPMERLTLEEHVEAKANTTYDITTSNRNMDKWTTWRAPK
AEF12557 MDHGDRLMSWKGREQWVKVQMEEKEPLLKEQDKGKYTRKGIQDFGYSPELPKGDNNNGDKTRRRRRRNRGWWKQLREIMQTRRANTTNDYSRSLEQCCGAMEQLTMEKHLETEANTTPSVASNSGTMDKWKNWRTPQK
ACV53616 MDKKDGKRTRTEEPPLREVWRQVMSEYRSRYPELQATEELAVSDHRTGPKGEGYRMRGRRRRRGRGWFRWLRKLRARRFTEPDPDLEDPVPGMETLTLGDPGEGADTDGAAGDSDGAVAAGLWTAWRSPQK
ACN82426 MDMGAKHMQRTGEGNWVEVKMEEQEREEEPLLHTRQQGIQDTKYPKIPKSYSDNGNKSRRGRRKRAGFWKWLRGIRNQQRAAKSNNQESMEQCVGALGNLTLGRAMEKEPSEAFNPPPNNGNMDKWTTWRKAQK
AAB31026 MDAGARYMRLTGKENWVEVTMDGEKERKREGFTAGQQDIQNSKYPDIPTGHSHHGNKSRRRRRKSGFWRWLRGIRQQRNKRKSDSTESLEPCLGALAELTLEGAMEKGPAEAARPSADDGNLDKWMAWRTPQK
ACA81612 MDRRLRKQWMEGVQAYKEFQADEESGVSDHRSGLKGEGYRLRGRRRRRRGWYRWLRKLRARRFTEPDPDLEDPIPDVEALTLDDRGEGADAADLATRGAGAVVADVWMEWRSPQKEKEKGGL
AAG48633 MAEIRKEAKEPLIQDQVSTSQLGDGDPGATRRRRRRRRKGWWRWLREMQRSRQQRNYERLEESLGDLEKLTLAEHMEECGGGAVGDSADNQNLAPWREWREPLKEK

B. Betaretroviruses

HERV-K

AAQ76762 MNPSEMQRKAPPRRRRHRNRAPLTHKMNKMVTSEEQMKLPSTKKAEPPTWAQLKKLTQLATKYLENTKVTQTPESMLLAALMIVSMVSAGVPNSSEETATIENGP

JSRV

Hofacre *et al*, 2009 MPKRRAGFRKGWYARQRNSLTHQMQRMTLSEPTSELPTQRQIEALMRYAWNEAHVQPPVTPTNILIMLLLLLQRVQNGAAAAFW

MMTV*

ABB02515 MPNHQSGSPTGSSDLLLSGKKQRPHLALRRKRRREMRKINRKVRRMNLAPIKEKTAWQHLQALIFEAEEVPKTSQTPQTSLTLFLALLSVLGPPPVTGESYWAYLPKPPILHPVGWGNTDPIRVLTNQTIYLGGSPDFHGFRNMSGNVHFEGKSDT

C. Deltaretroviruses

BLV

ACR15159 MPKERRSRRRPQPIIRWRQNYFLSFKQVLLVGGPTLYMPARPWFCPMTSPSMPGAPSAGPMSDSNSKGSTPRPPARPTVSPGPPMDDLSASMERCSLDCMSPRPAPKGPDDSGSTAPFRPFALSPARFHFPPSSGPPSSPTNANCPRPLATVAPSSGTAFFPGTT
AAF97919 MPKERRSRRRPQPIIRWRQNYFLSFKQVLLVGGPTLYMPARPWFCPMTSPSMPGAPSAGPMSDSNSKGSTPRTPARPTVSPGPPMDDLSASMERCSLDCMSPETRPQGPDDSGSTAPFRPFALSPARFHFPPSSGPPSSPTNANCPRPLATVAPSSGTAFFPGTT

PTLVs

AAA85844 MPKTRRRPRRSQRKRPPTPWPTSQGLDRVFFSDTQSTCLETVYKATGAPSLGDYVRPAYIVTPYWPPVQSIRSPGTPSMDALSAQLYSSLSLDSPPSPPREPLRPSRSLPRQSLIQPPTFHPPSSRPCANTPPSEMDTWNPPLGSTSQPCLFQTPDSGPKTCTPSGEAPLSACTSTSFPPPSPGPSCPT
AAB59886 MPKTRRQRTRRARRNRPPTPWPISQDLDRASYMDTPSTCLAIVYRPIGVPSQVVYVPPAYIDMPSWPPVQSTNSPGTPSMDALSALLSNTLSLASPPSPPREPQGPSRSLPLPPLLSPPRFHLPSFNQCESTPPTEMDAWNQPSGISSPPSPSPNLASVPKTSTPPGEKP
AAD34844 MPKTRRQRTRRARRNRPPTPWPISQDLDRASYMDTPSTCLAIVYRPIGVPSQVVYVPPAYIDMPSWPPVQSTNSPGTPSMDALSALLSNTLSLASPPSPPREPQGPSRSLPLPPLLSPPRFHLPSFNQCESTPPTEMDAWNQPSGISSPPSPSLNLASVPKTSTPPGEKP
AAG48704 MPKTRRQRTRRARRNRPPTPWAISQDLDRASYMDTPSTCLAIVYRPIGVPSQVVYVPPAYIDMPSWPPVQSTNSPGTPSMDALSALLSNTLSLASPPSPPREPQGPSRSLPLPPLLSPPRFHLPSFNQCESTPPTEMDAWNQPSGISSPPSPSLNLASVPKTSTPPGEKP
AAG48730 MPKTRRQRTRRARRNRPPTPISQDLDRASYMDTPSTCLAIVYRPIGVPSQVVYVPPAYIDMPSWPPVQSTNSPGTPSMDALSALLSNTLSLASPPSPPREPQGPSRSLPLPPLLSPPRFHLPSFNQCESTPPTEMDAWNQPSGISSPPSPSLNLASVPKTSTPPGEKP
ADQ00640 MPKTRRQRTRRARRNRPPTPWPISQDLDRASYIDTPSTCLAIVYRPIGVPSQVVYVPPAYIDMPSWPPVQSTSSPGTPSMDALSALLSNTLSLASPPSPPREPPRPSRSLPLPPLLSPPRFHPPSSNQCENTPPIAMDAWNQPSGISSPPSPSLNLASVPKTSTPPGEKP
AAZ77660 MPISQVSDRAFSTGTLSTFSATVYRPIGAPFLGGFVPLGYTAMPYWPRAPNIRLPGTPSMDALSAQLYNTLSLDSPPSPPRELPAPSRFSPPQPLLRPPRFLHPSSTPLKNTPPSETIALNSPWESSCQPCPSPTLGSDPKTSTPCGEAPLCAFTSISSPPP
ABF18962 MPKTRKQRSRRPRNQRPSTPWPISQVSDRAFSTGTLSTFSATVYRPIGAPFLGGFVPLGYTAMPCWPRAPNIRLPGTPSMDALSAQLYNTLSLGSPPSPPKELPAPSRFSPPQPLLRPPRFLHPSSTPLKNTPPSETIASSSPWESSCQPCPSPTLGSGPKTSTPYGAAPSCVSTSISSPPP
AAU34011 MPKTRRGHRRSQRKRPPTPWPISQGLDKASSMDTQSTCLETVCRATGAPSLGDYAQPAFIVMPFWPLARNTRSPGTPSMDALSDQLYNSLSLDSPLSPPSEPPRPLKSSPLQPLIRPPTFRPPSSKPCASTPRFETDIWSPPLESNSRPCLSPTPAYVPKTSIPSGEIQSSASTSTNYPPQSPGPSCLM
ACF40917 MPKTRKQRSRRPRNQRPSTPWPISQVSDRAFSTGTLSTFSATVYRPIGAPFLGGFVPLGYTAMPCWPRAPNIRLPGTPSMDALSAQLYNTLSLGSPPSPPKELPAPSRFSPPQLLLRPPRFLHPSSTPLKNTPPSETIASSSPWESSCQPCPSPTLGSGPKTSTPYGAAPSCVSTSISSPPP
AAN87147 MPKTRKQRSRRPKNQRPSTPWPISQVSGRAFSTGTLSTFSATVYRPIGAPFLGGFVPLGYTAMPCWPRAPNIRLPGTPSMDALSAQLYNTLSLDSPPSPPRELPAPSRFSPPQLLLRPPRFLHPSSTQSKNTPPSEIIASSSPWENSCQPCPSPTLDSDPKTSTPCGEAPLCAFTSISSPPP
AAO86628 MPKTRKQRSRRPKNQRPSTPWPISQVSGRAFSTGTLSTFSATVYRPIGAPFLGGFVPLGYTAMPCWPRAPNIRLPGTPSMDALSAQLYGTLSLDSPPSPPRELPAPSKFSPPQLLLQPPRFLHPSSTQSENTPLSETTASSSPWESSCQPCPSPTLGSDPKTSTPCGEAPLCACTCISSPPP
AAO62105 MPKTRKQRSRRPKNQRPSTPWPTSQVSGRAFSTGTLSTFSATVYRPIGAPFLEGFVPLGYTAMPCWPRAPNIRLPGTPSMDALSARLYNTLSLDSPPSPPKELPAPSRFSPPQPLLRPPRFLHLSSTQLENTPPSETIASSSPWGSSCPPCPSPTPGFDPKTYTPCGAAPSCASTSTSSPPP
